# A low-input high resolution sequential chromatin immunoprecipitation method captures genome-wide dynamics of bivalent chromatin

**DOI:** 10.1186/s13072-024-00527-9

**Published:** 2024-02-10

**Authors:** Janith A. Seneviratne, William W. H. Ho, Eleanor Glancy, Melanie A. Eckersley-Maslin

**Affiliations:** 1 Peter MacCallum Cancer Centre, Melbourne, Victoria 3000 Australia; 2 Sir Peter MacCallum Department of Oncology, The University of Melbourne, Victoria, 3010 Australia; 3 Department of Anatomy and Physiology, The University of Melbourne, Victoria, 3010 Australia

**Keywords:** Bivalent chromatin, Embryonic stem cells, Plasticity, Bivalency, Chromatin immunoprecipitation, Epigenetics, H3K4me3, H3K27me3, Sequential ChIP, ChIP-reChIP

## Abstract

**Background:**

Bivalent chromatin is an exemplar of epigenetic plasticity. This co-occurrence of active-associated H3K4me3 and inactive-associated H3K27me3 histone modifications on opposite tails of the same nucleosome occurs predominantly at promoters that are poised for future transcriptional upregulation or terminal silencing. We know little of the dynamics, resolution, and regulation of this chromatin state outside of embryonic stem cells where it was first described. This is partly due to the technical challenges distinguishing bone-fide bivalent chromatin, where both marks are on the same nucleosome, from allelic or sample heterogeneity where there is a mix of H3K4me3-only and H3K27me3-only mononucleosomes.

**Results:**

Here, we present a robust and sensitive method to accurately map bivalent chromatin genome-wide, along with controls, from as little as 2 million cells. We optimized and refined the sequential ChIP protocol which uses two sequential overnight immunoprecipitation reactions to robustly purify nucleosomes that are truly bivalent and contain both H3K4me3 and H3K27me3 modifications. Our method generates high quality genome-wide maps with strong peak enrichment and low background, which can be analyzed using standard bioinformatic packages. Using this method, we detect 8,789 bivalent regions in mouse embryonic stem cells corresponding to 3,918 predominantly CpG rich and developmentally regulated gene promoters. Furthermore, profiling Dppa2/4 knockout mouse embryonic stem cells, which lose both H3K4me3 and H3K27me3 at approximately 10% of bivalent promoters, demonstrated the ability of our method to capture bivalent chromatin dynamics.

**Conclusions:**

Our optimized sequential reChIP method enables high-resolution genome-wide assessment of bivalent chromatin together with all required controls in as little as 2 million cells. We share a detailed protocol and guidelines that will enable bivalent chromatin landscapes to be generated in a range of cellular contexts, greatly enhancing our understanding of bivalent chromatin and epigenetic plasticity beyond embryonic stem cells.

**Supplementary Information:**

The online version contains supplementary material available at 10.1186/s13072-024-00527-9.

## Background

The chromatin landscape of cells not only shapes cellular identity, but also enables how cells are able to respond and adapt to a changing environment. Amongst the multitude of different layers of organisation, histone post-translational modifications are tightly associated with the activity and accessibility of the underlying DNA sequence. In particular, tri-methylation of lysine 4 on histone 3 (H3K4me3) is tightly correlated with active promoters, whilst tri-methylation of lysine 27 of histone 3 (H3K27me3) is associated with heterochromatin and gene repression [1–3]. Remarkably these two seemingly opposing histone modifications can be found on opposite tails of the same nucleosome where it is thought to reflect a poised state of the underlying DNA sequence that is amenable to future activation or repression (reviewed in [4, 5]). In mouse embryonic stem cells, removing bivalent chromatin results in the accumulation of tightly repressive DNA methylation and the inability of the genes to be activated in a timely manner upon differentiation [6–10]. Therefore, bivalent chromatin is a classic exemplar of molecular plasticity, by priming genes for the future and facilitating cell adaptation. However, bivalent chromatin has been predominantly studied in the context of mouse embryonic stem cells (ESC) where it was first described [11, 12]. This is partly due to technical challenges associated with accurately detecting this important structure. Consequently, our current understanding of the distribution and dynamics in other cell types and species remains limited.

**Fig. 1:**
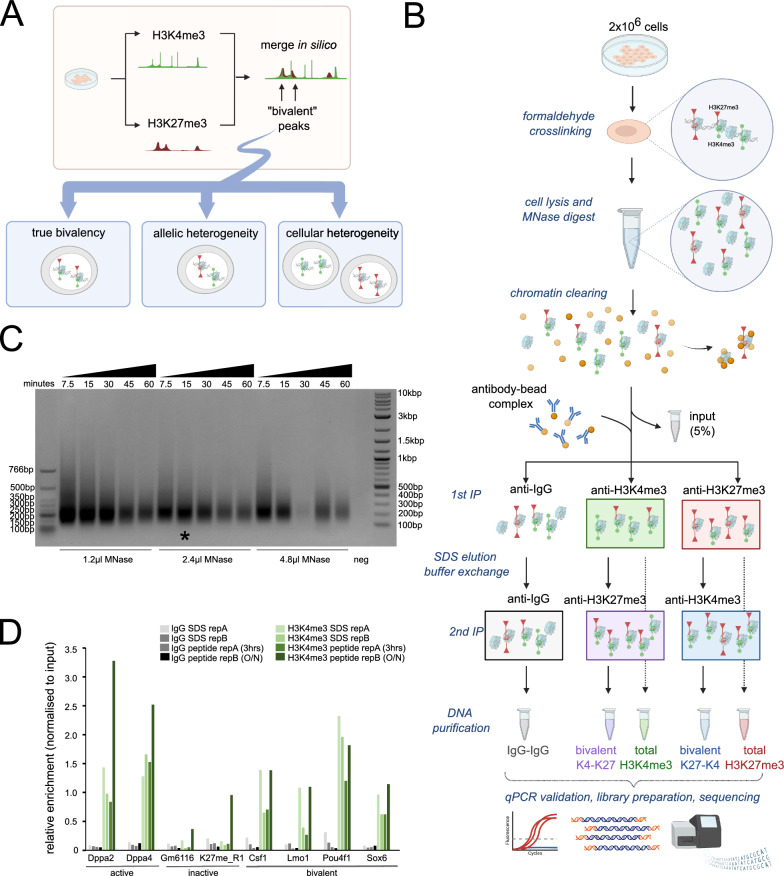
Development of an optimised ChIP-reChIP protocol to robustly measure bivalent chromatin. **A** Potential limitations in using independent total H3K4me3 (green, circles) and total H3K27me3 (red, triangles) datasets in distinguishing bone-fide bivalent chromatin, where the two marks occur on the same nucleosome, from allelic and cellular heterogeneity. **B** overview of sequential ChIP-reChIP protocol, see methods for detailed description. **C** Agarose gel showing chromatin digested with 1.2μl, 2.4μl or 4.8μl MNase for different amounts of time (7.5 min to 60 min). 2 million cells were used per condition. Negative control did not have any MNase added. Star denotes final condition used in protocol. **D** Single H3K4me3 (green) and IgG control (grey) ChIP-qPCR analysis comparing SDS-based elution (light) from peptide elution (dark). Two active H3K4me3-only (Dppa2, Dppa4), two inactive H3K27me3-only (Gm6116, K27me_R1) and four bivalently marked loci (Csf1, Lmo1, Pou4f1, Sox6) were analyzed. Enrichment values normalised to input are shown

A major challenge in studying bivalent chromatin is that the co-occurrence of active H3K4me3 and inactive H3K27me3 histone modifications needs to be distinguished from instances where the histone modifications occur on different alleles in the cell or in different cells within a mixed population (Fig. 1A). Consequently, performing independent chromatin immunoprecipitation (ChIP) or CUT&RUN-related methods separately for H3K4me3 and H3K27me3 and then overlapping peaks in silico is not sufficient to be absolutely certain the region is indeed bivalent and not a consequence of allelic or cellular heterogeneity. This becomes even more of a challenge when analysing complex systems such as developing tissues or patient cancer samples. Previous studies in human T cells and mouse ESCs have suggested that as many as 14% to 25%, respectively, of bivalent regions called using independent ChIPs are false-positives [13, 14]. To address this, sequential ChIP or ChIP-reChIP approaches have been developed [13–18], whereby the chromatin purified from a first immunoprecipitation reaction (e.g., H3K4me3) is used as input into a second immunoprecipitation reaction for a different modification (e.g., H3K27me3). Theoretically, only chromatin with both marks of interest are purified in this way. However, these protocols typically require tens of millions of cells as input and so are not always feasible and often lack appropriate controls, leading to many false-positives. Moreover, poor signal-to-noise makes data interpretation and downstream analysis complex. Recently, multi-tagmentation methods have been described to simultaneously map multiple histone modifications in single-cells [19–21], yet these methods required custom reagents such as different barcoded Tn5 complexes or nanobodies, and complex data-analysis pipelines. Therefore, there is a need for sensitive, robust and cost-effective methods to accurately detect bivalent chromatin in low cell numbers that can use existing standardised downstream data-analysis approaches.

Here, we present a highly-optimized sequential ChIP (reChIP) methodology for accurately detecting bivalent chromatin along with controls from just 2 million cells. From one sample, our refined method generates 5 datasets including the 2 reChIP datasets (H3K4me3 followed by H3K27me3 and vice versa) and 3 control datasets (IgG–IgG background reChIP, in-line total H3K4me3 and in-line total H3K27me3). By applying our method in mouse ESCs we detected 8,789 bivalent chromatin regions which occurred predominantly at CpG-rich promoters. Importantly, in addition to 77% of previously annotated bivalent genes [14], our method revealed an additional 1,511 bivalent gene promoters in mouse ESCs. Lastly, we validated the sensitivity of our method by profiling ESCs lacking the epigenetic priming factors *Dppa2* and *Dppa4* which are required for maintaining bivalent chromatin at a subset of promoters [6, 7]. This confirmed the ability of our method to detect dynamic changes in bivalent chromatin. In summary, our method provides a much-needed resource for researchers wishing to accurately map bivalent chromatin landscapes from as little as 2 million cells.

## Results

### Development of an optimised ChIP-reChIP protocol to robustly measure bivalent chromatin

A challenge in studying bivalent chromatin is that aligning independently generated single H3K4me3 and H3K27me3 datasets in silico is theoretically insufficient to distinguish true bivalency (where both marks are present on the same chromatin fragment) from allelic or cellular heterogeneity (where marks are present on different alleles or in different cells within the population) (Fig. 1A). To address this, reChIP (also known as sequential ChIP or ChIP-reChIP) approaches have been used [13–18, 22, 23], however, many limitations exist with current protocols which typically require large (> 10 million cells) amount of starting material per reaction (13–15, 22, 23), and consequently are often performed in just one of the two directions [23]. This is a major issue as many false positives can confound the results due to “signal carry-over” whereby enrichment from the first ChIP carries through into the second ChIP. Moreover, variable data quality with low signal to noise has traditionally made downstream bioinformatic analysis of bivalent regions challenging. To address these points, we optimised the reChIP protocol to give a high signal to noise ratio with both qPCR and high-throughput sequencing readouts from just 2 million cells (Fig. 1B). Critically, we advise the reChIP be carried out in both orientations (H3K4me3 followed by H3K27me3 and vice versa). The method was optimised using serum/LIF cultured E14 mouse Embryonic Stem Cells (ESCs) given their well-defined distribution of bivalent chromatin [11, 12, 14, 18]. Importantly our method produces high quality data that can be analysed with commonly-used bioinformatic tools. A full detailed protocol accompanies this paper (Additional file 1).

The 3-day workflow is shown in Fig. 1B. Briefly, cells are treated with formaldehyde to cross-link chromatin, and 2 million cell aliquots can then be stored at − 80 ℃ for up to 6 months. Cells are gently lysed and treated with MNAse to generate predominantly mononucleosomes (Fig. 1C, Additional file 2: Figure S1A) and chromatin pre-cleared by incubating with pre-washed dynabeads for 3 h at 4 ℃ to reduce non-specific binding. Alternatively, sonication can be used to fragment chromatin with similar results (Additional file 2: Figure S1A, B). During the pre-clearing step, the antibody-dynabead complexes are formed for the IgG control, H3K4me3 and H3K27me3 immunoprecipitations. 5% of the precleared chromatin is set aside as an input control and the remainder split across the three tubes of antibody-dynabead complexes for the first overnight immunoprecipitation at 4 ℃.

Following the first immunoprecipitation, the chromatin-antibody-dynabead complexes are thoroughly washed to remove any non-specific binding prior to chromatin elution. Traditionally, reChIP protocols typically use one of two approaches to elute chromatin from beads. DTT- or SDS-based elution buffers function by dissociating the affinity interactions upon which the immunoprecipitation is based but require additional dilution and/or cleanup steps to ensure compatibility with a second immunoprecipitation reaction (14–17, 22, 23). An alternative is to use high concentration of modified histone tail peptides to compete with antibody binding sites [13]. We compared these two approaches to elute chromatin in single H3K4me3 ChIPs (Fig. 1D). The SDS elution performed well in terms of specificity and signal to noise ratio. The 3 hour peptide competition gave similar results to SDS elution, however, the amount of unspecific background signal increased when incubated overnight (Fig. 1D). After considering costs and availability of commercial peptides, we decided to implement SDS elution in our final protocol. To facilitate subsequent antibody binding events, we diluted the chromatin and performed a buffer exchange using 3 kDa molecular weight filters. From the first immunoprecipitation reaction, 10% of the sample representing in-line total H3K4me3 or total H3K27me3 control can be set aside to aid downstream analysis and bivalent peak classification, although we recommend performing independent total H3K4me3 and H3K27me3 ChIPs when possible. The second immunoprecipitation is then performed overnight using the alternate antibody so that the reChIP is performed in both directions: H3K4me3 followed by H3K27me3 (K4-K27), and H3K27me3 followed by H3K4me3 (K27-K4). As a negative control, IgG followed by IgG (IgG–IgG) is also performed to control for non-specific enrichment during the reChIP assay. Chromatin is then eluted in SDS-elution buffer, formaldehyde crosslinks reversed, RNA and proteins degraded, and enriched DNA fragments purified ready to be processed for qPCR analysis and/or high throughput sequencing.

### Generating high quality genome-wide bivalent chromatin maps in mouse embryonic stem cells

**Fig. 2:**
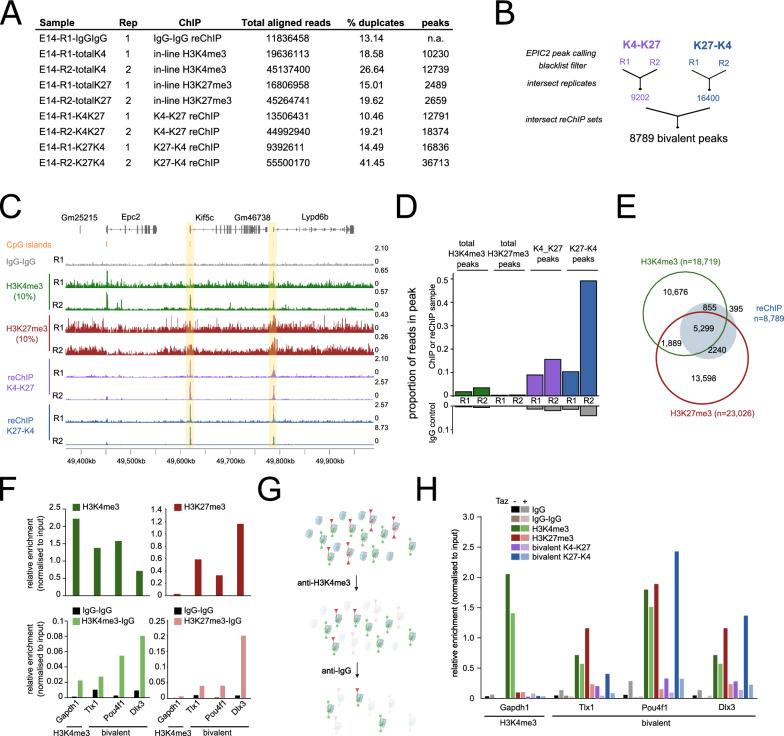
**A** Summary table of samples sequenced in E14 mouse embryonic stem cells indicating replicate (Rep), ChIP, total aligned reads, percentage duplication and number of peaks. **B** Analysis pipeline for calling bivalent peaks. **C** Genome browser view of reChIP datasets including IgG–IgG reChIP control (grey), in-line total H3K4me3 (green, rows 2 and 3), in-line total H3K27me3 (red, rows 4 and 5) and bivalent reChIP for H3K4me3 followed by H3K27me3 (K4-K27, purple, rows 6 and 7) or vice-versa (K27-K4, blue, row 8 and 9). Two biological duplicates (R1 and R2) are shown for all but IgG–IgG libraries. CpG islands are denoted by orange bars. Bivalent regions are highlighted in yellow. **D** FRiP scores showing proportion of reads within peaks for each individual sample. IgG–IgG (grey) is shown for each set of peaks to get background levels. **E** Comparison of peaks called using our reChIP method compared to in silico overlap of independently derived total ChIP-seq datasets **F** Single ChIP-qPCR for H3K4me3 (green) or H3K27me3 (red) at a H3K4me3 region (Gapdh1) or three bivalent regions (Tlx1, Pou4f1, Dlx3). Bottom panel shows corresponding reChIP controls: IgG–IgG (black), H3K4me3-IgG (light green), H3K27me3-IgG (light red). **G** Schematic of enrichment of first IP into the second IP when IgG antibody is used as the second IP in reChIP experiments. **H** reChIP-qPCR analysis of a H3K4me3-only region (Gapdh1) and three bivalent regions (Tlx1, Pou4f1 and Dlx3) in control (dark bars) versus cells treated with Tazemetostat (Taz) to reduce global H3K27me3 levels (light bars)

To date bivalent chromatin is best understood in mouse ESCs. Therefore, we used this model to test our refined method. In total 9 datasets were generated from two biological replicates (Fig. 2A). These included two in-line H3K4me3 single ChIPs, two in-line H3K27me3 single ChIPs, two each of K4-K27 and K27-K4 reChIPs, and one IgG–IgG replicate. We note that the widely-used commercial ChIP-grade H3K4me3 antibody used predominantly in our manuscript has also been reported to detect H3K4me2 at lower efficiency [24], and so we may also be detecting some H3K4me2-H3K27me3 bivalent nucleosomes. Replicate 2 was sequenced at a higher depth (45–55 million reads per sample) than replicate 1 (9.4–19.6 million reads per sample), to enable us to determine optimal library sequencing depth through downsampling analysis (see below).

The bioinformatic workflow for calling bivalent peaks is outlined in Fig. 2B. Initial data inspection of our reChIP datasets revealed strong peak distribution of reads for the K4-K27 and K27-K4 reChIP samples at known bivalent regions with low intervening background signal (Fig. 2C). Furthermore, peaks were observed in the in-line total H3K4me3 and total H3K27me3 samples, albeit these signals were noisier. This is likely due to the lower starting material for library preparation of these samples which only correspond to approximately 60,000 cells. This is near the lower limit for achieving clear signal over background for single ChIPs in our laboratory (Additional file 2: Figure S1C).

We sequenced the two biological replicates at different depths ranging from approximately 10 million through to 55 million aligned reads (Fig. 2A). From the higher coverage replicate 2, we performed in silico downsampling analysis from which we concluded that 15–20 million reads was a good compromise between number of high-confidence bivalent peaks and promoters detected (see below) versus sequencing cost (Additional file 2: Figure S1D, E). Supporting this, even with approximately 10 million mapped reads in replicate 1 there were clear peaks in the reChIP samples (Fig. 2C). Moreover, downsampling independently derived total H3K4me3 and H3K27me3 datasets generated from approximately 10 million cells had no negligible effect on the number of peaks called (Additional file 2: Figure S1F, G), suggesting it is the starting cell number, not the sequencing depth, that dictates data quality.

To get a measure of the specificity of our assay, we calculated the fraction of reads in called peak regions (FRiP score) which is commonly used in ATAC-seq analysis to determine library quality. Notably, all reChIP samples had very high FRiP scores, while the corresponding IgG–IgG scores for each of the peak sets were all less than 0.1 (Fig. 2D). This indicates a very high and specific enrichment and low background of these reChIP libraries.

Overlapping total H3K4me3 and total H3K27me3 peaks in silico is often used as a proxy to define bivalent chromatin (Fig. 1A). Using this approach with the independent 10 million cell datasets gives 7,188 putative bivalent regions, of which 5,299 (73.7%) overlapped a peak in both reChIP datasets (Fig. 2E). Crucially, 1,889 in silico called peaks are not validated in both reChIP directions indicating a high false-positive rate of 26.3% (Fig. 2E). This may be explained by allelic or cellular heterogeneity in mouse embryonic stem cells and highlights the importance of performing reChIP experiments to accurately measure bivalent chromatin.

A frequently used control in sequential ChIP experiments is to use IgG as a second antibody [14, 22]. The rational for this is that the IgG should not enrich for any chromatin regions and thus the H3K4me3-IgG and/or H3K27me3-IgG reChIPs can be used to estimate non-specific enrichment levels and control for signal carry-through from the first ChIP into the second. To test the utility of these controls we performed H3K4me3-IgG and H3K27me3-IgG reChIP-qPCRs in mESCs (Fig. 2F). However, these reChIPs still showed substantial enrichment at bivalent regions, while the IgG–IgG reChIPs did not enrich at any region (Fig. 2F). The enrichment at H3K4me3-IgG and H3K27me3-IgG reChIPs had a near identical enrichment pattern to the corresponding total H3K4me3 or H3K27me3 single ChIPs. Consistently, reanalysis of published reChIP data in mESCs [14] showed near identical enrichment between total H3K4me3 and H3K4me3-IgG reChIP datasets (Additional file 2: Figure S1H). This suggests that the use of IgG as a secondary ChIP is subsampling the first enrichment (Fig. 2G), and so not a useful negative control.

As an alternative control for signal carry-over from the first ChIP into the second, we treated mESCs with the Ezh2 inhibitor Tazemetostat to deplete global levels of H3K27me3 [25, 26]. As expected, single ChIP-qPCR revealed total H3K27me3 at these regions was completely lost in the Tazemetostat-treated cells, while total H3K4me3 was unaffected (Fig. 2H). Importantly, in Tazemetostat-treated cells, the reChIP signal in both orientations was also depleted. This indicates that our reChIP protocol is specific for detecting *bone fide* bivalent chromatin and not affected by signal from the first ChIP carrying through into the second.

### Identification of 8,789 bivalent peaks in mESCs

**Fig. 3:**
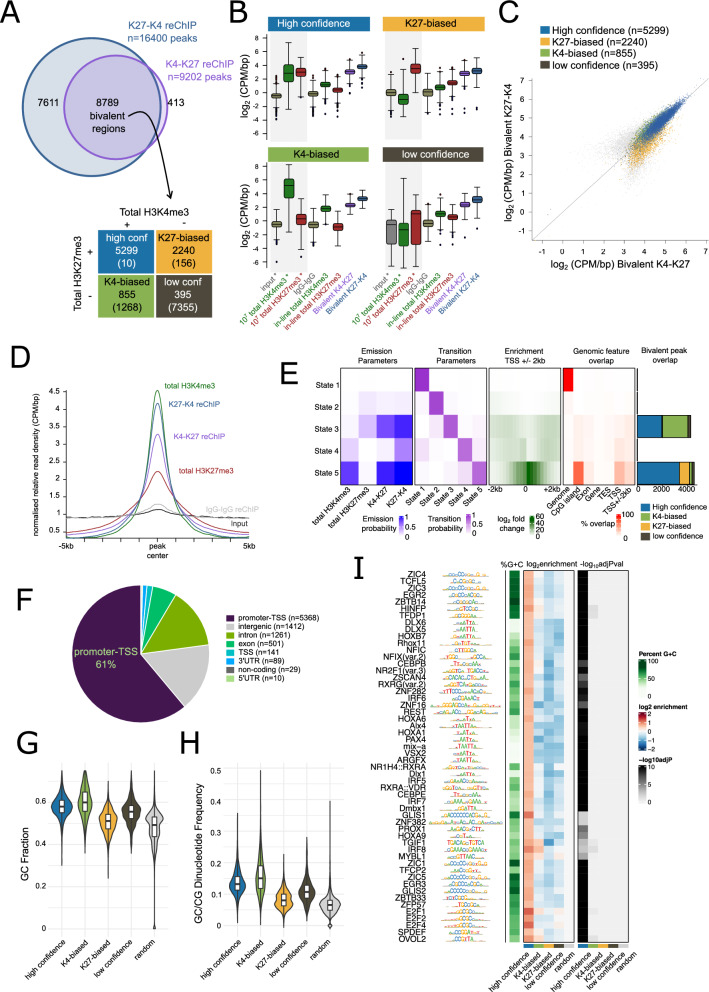
Identification of 8,789 bivalent regions in mouse embryonic stem cells. **A** Overlap between K27-K4 (blue) and K4-K27 (purple) reChIP datasets. The 8,789 overlapping peaks were classified as high confidence (overlap peak in both total H3K4me3 and total H3K27me3, blue), K4-biased (overlap peak in only total H3K4me3, green), K27-biased (overlap peak in only H3K27me3, orange) or low confidence (does not overlap peak in either H3K4me3 or H3K27me3, brown) using independent total H3K4me3 and H3K27me3 single ChIPs from approximately 10 million cells from GSE135841 [7]. **B** Box-whisker plots (line shows median and box denotes 25th and 75th percentile, whiskers shown interquartile range multiplied by 2) showing log_2_CPM/bp values for high confidence (top left), K4-biased (top right), K27-biased (bottom left) and low confidence (bottom right) peaks in independent total H3K4me3 and total H3K27me3 datasets from GSE135841 [7] (denoted by * and shaded grey background) or the in-line total and reChIP datasets generated in this study. **C** Scatter plot showing log_2_CPM/bp values for bivalent K4-K27 (x-axis) and bivalent K27-K4 (y-axis) datasets for all bivalent peaks highlighting high confidence (blue), K4-biased (green), K27-biased (orange) and low confidence (brown) peaks. **D** Relative distribution plot (each probe is weighted equally in final profile) of reads across peaks showing broader peak width for H3K27me3 peaks compared to H3K4me3 and reChIP peaks **E** 5-state chromHMM models using pooled replicates for in-line total H3K4me3, in-line total H3K27me3 and K4-K27 and K27-K4 reChIP datasets showing emission (left) and transmission (second from left) parameters, enrichment across TSS ± 2kb and overlap with high confidence (blue), K4-biased (green), K27-biased (orange) and low-confidence (brown) bivalent regions (right). **F** Genomic features associated with the bivalent regions. **G**, **H** Violin plots showing GC fraction **G** and GC-CG dinucleotide frequency **H** within regions compared to random subset (width and GC-content matched) of 8,789 genomic regions. All comparisons are statistically significant after multiple testing (Benjamini–Hochberg correction). **I** Motif enrichment for the four classes of bivalent peaks compared to the same background set used in **G**, **H**. Those with log_2_enrichment over random sequences > 1 are shown, along with their enrichment scores and -log_10_Adjusted p-value

In our mESC datasets, we revealed 9,202 K4-K27 and 16,400 K27-K4 reChIP peaks of which 8,789 were shared (Fig. 3A). We first investigated whether these peaks were similarly shared in total H3K4me3 and/or total H3K27me3 datasets. We initially stratified bivalent peaks using our in-line total H3K4me3 and H3K27me3 single ChIPs. We were unable to call many peaks using these in-line controls (Additional file 2: Figure S2A), although enrichment for total H3K4me3 and total H3K27me3 at bivalent regions was still observed (Fig. 3B). This is likely due to the low signal- to-noise seen in these datasets that correspond to approximately 60,000 cells (Fig. 2D). Therefore, as an alternative we stratified the reChIP peaks using independent total ChIPs from approximately 10 million cells we previously generated using the same cell line [7]. When using these independently derived total ChIP-seq datasets, 5,299 of the 8,789 peaks (60%) overlapped peaks in both the total H3K4me3 and H3K27me3 datasets (Fig. 3A). We termed these high-confidence promoters to distinguish them from K4-biased (peak shared only in total H3K4me3, n = 855), K27-biased (peaks shared only in total H3K27me3, n = 2,240) or low confidence (did not share a peak in either total H3K4me3 or H3K27me3, n = 395) bivalent regions. As expected, high confidence bivalent regions had the highest enrichment in reChIP datasets (Fig. 3B, C). Representative H3K4me3-only, high-confidence, K4-biased, K27-biased and low-confidence bivalent regions are shown in Additional file 2: Figure S2B-F.

The bivalent reChIP peaks were sharp and narrow (Fig. 3D), demonstrating the specificity of our approach in enriching for chromatin fragments containing both modifications of interest, and the absence of carry-over of the broader H3K27me3 signal, particularly in the K27-K4 reChIP dataset. Orthogonal unbiased Hidden Markov Model approaches [27] using in-line totals and reChIPs identified chromatin states that matched our peak-centric classifications (Fig. 3E). From the 5-state chromatin model we observed that state 3 and state 5 were characterised by high signal in both K4-K27 and K27-K4 datasets, as well as in-line controls. Both these states were enriched for our bivalent promoters, confirming the validity of these bivalent peak subclasses.

Consistent with previous studies [4, 5, 11], bivalent peaks were predominantly located at promoters or transcription start sites (TSS) (80%) (Fig. 3F) which contained CpG islands (Additional file 2: Figure S2G). Sequence analysis revealed that bivalent regions had a higher GC content (Fig. 3G) and CpG dinucleotide frequency (Fig. 3H) than size and GC-content matched random set of genomic regions. Motif analysis revealed a strong enrichment for motifs associated with developmental regulation including ZSCAN4, HOX(A1,A6,A9,B7), PROX1, TGIF1, PAX4/9 and ZIC1/4/5 (Fig. 3I) in the high confidence bivalent regions. Approximately half (51.8%) of non-promoter bivalent peaks predominantly overlapped candidate enhancer elements (Additional file 2: Figure S2H), suggesting a potential regulatory role for this chromatin state at enhancer elements.

### Catalogue of 3,918 high-confidence bivalent gene promoters in mouse embryonic stem cells

**Fig. 4:**
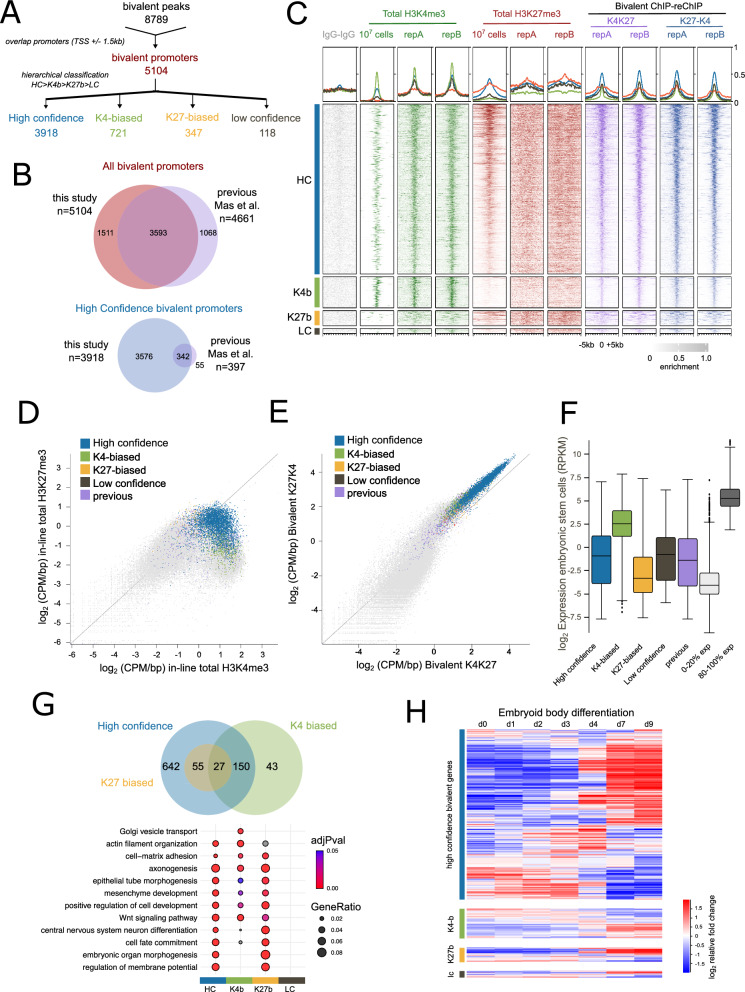
Catalogue of 5,104 bivalent gene promoters in mouse embryonic stem cells. **A** Schematic outlining classification strategy for bivalent promoters. Full list of bivalent promoter classifications is available in Additional file 3: Table S3. **B** Overlap of all bivalent promoters (top) and high-confidence bivalent promoters (bottom) between those identified in this study compared to previously published reChIP data (reanalyzed from [14]). **C** Enrichment heatmaps showing CPM/bp normalised read densities for high confidence (top row), K4-biased (second row), K27-biased (third row) and low confidence (bottom row) bivalent promoters after scaling for all datasets analysed. Peaks were extended to 5kb upstream and downstream of the peak centre. Values surpassing the 99th percentile have been masked for visualisation. 10^7^ samples refers to independent total H3K4me3 and total H3K27me3 datasets from GSE135841 [7] **D, E** Scatterplot showing log_2_enrichment (CPM/bp) of **D** in-line total H3K4me3 (x-axis) and in-line total H3K27me3 (y-axis) or **E** bivalent K4-K27 (x-axis) and K27-K4 (y-axis) reChIP datasets for all promoters highlighting those that overlap different classes of bivalent peaks defined using independent 10 million cell total H3K4me3 and total H3K27me3. **F** Box plot showing log_2_ RPKM gene expression levels in mouse embryonic stem cells for four different classes of bivalent genes and those redefined from previous data (14). Expression of the bottom 20% and top 20% is shown as a comparison. Gene expression data reanalyzed from GSE135841. **G** Gene Ontology analysis showing overlap of representative enriched terms in the four classes of bivalent genes (top) and gene ratios and adjusted P-value of selected terms (bottom). The full list of enriched terms is available in Additional file 3: Table S4. **H** log_2_ fold change in gene expression levels for different classes of bivalent genes across 9 days of embryoid body differentiation. Each gene has been normalised separately across the time series, genes are grouped using correlation based clustering. Gene expression data reanalyzed from (GSE135841)

Given the strong overlap between high confidence bivalent regions and gene promoters, we next analysed gene promoters specifically and found a total of 5,104 bivalent gene promoters in mESCs (Fig. 4A). As some promoters contained more than one bivalent peak, to classify the promoters, we used a hierarchical classification approach whereby promoters were first classed as high confidence if there were any high confidence peaks, then K4-biased followed by K27-biased and lastly low confidence (Fig. 4A). The majority of bivalent promoters were high confidence using the independent 10 million cell totals for classification. To directly compare our list of bivalent promoters with previous studies [14], we re-processed and classified the previous datasets using our pipeline (Additional file 2: Figure S3A), identifying 4,661 bivalent promoters. Importantly, our method detected 3,593 (77%) of these previously annotated bivalent genes (Fig. 4B). Closer examination of the novel bivalent promoters captured with our improved method saw an enrichment of signal in previous reChIP datasets at these loci (Additional file 2: Figure S3B), suggesting that these bivalent regions are real and that the increased signal to noise with our method enables these novel bivalent promoters to be captured. Of note, however, is our improved sensitivity in detecting high-confidence bivalent gene promoters. With the previous dataset, only 397 bivalent promoters were classified as high confidence in contrast to the 3,918 in our study, highlighting the increased sensitivity of our method.

The high confidence bivalent gene promoters had the highest levels of total H3K4me3 and total H3K27me3 (Fig. 4C, D) and bivalent K4-K27 and K27-K4 reChIP enrichment (Fig. 4C, E). As expected, the high confidence bivalent genes were expressed at low yet detectable levels in pluripotent mouse embryonic stem cells (Fig. 4F). In contrast the K4-biased bivalent genes had higher expression values, consistent with their enrichment for total H3K4me3 but not total H3K27me3. The high confidence bivalent genes were enriched in developmental biological processes (Fig. 4G) of which many were shared with the K4-biased or K27-biased classes. In line with current models [4, 5, 28], high confidence bivalent genes were dynamically expressed upon differentiation resolving to either an active or repressed state (Fig. 4H, Additional file 2: Figure S3C). Therefore, our data support the current model of bivalent chromatin marking developmental genes in embryonic stem cells, that are poised for future activation or repression.

### Profiling bivalent chromatin dynamics in DPPA2/4 knockout mouse embryonic stem cells

**Fig. 5:**
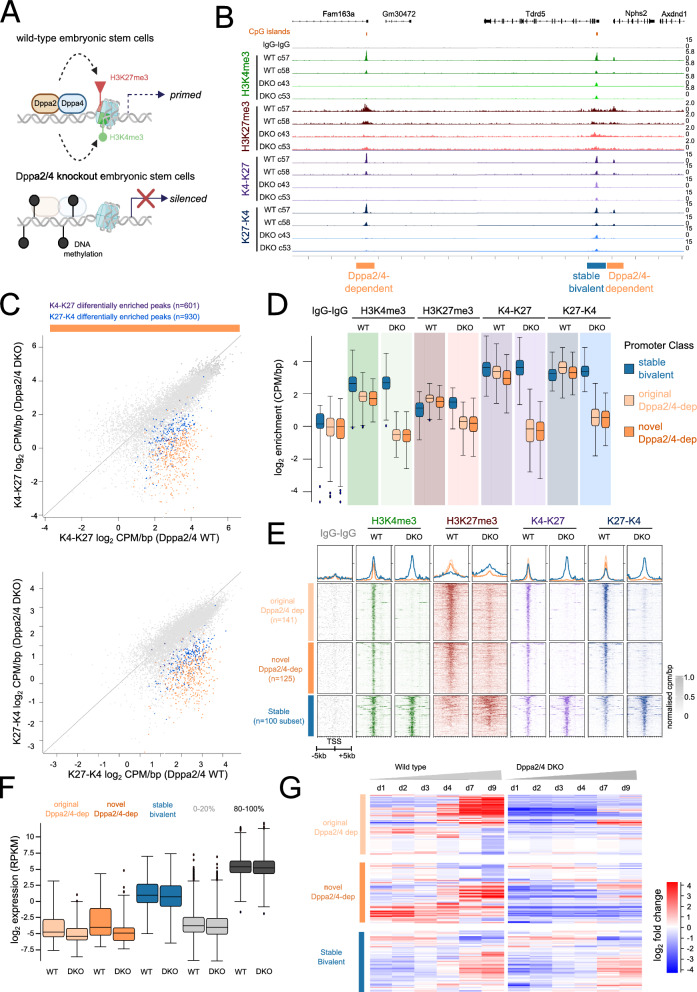
Profiling bivalent chromatin dynamics in DPPA2/4 knockout mouse embryonic stem cells. **A** Schematic depicting how DPPA2/4 maintain both H3K4me3 and H3K27me3 at a subset of bivalent genes, priming them for future activation. Loss of DPPA2/4 leads results in loss of both H3K4me3 and H3K27me3 and gain of repressive DNA methylation (black circles). **B** Genome browser view of wild type (WT, dark) and DPPA2/4 double knockout (DKO, light) embryonic stem cell clones. Two clones of each genotype are shown. In-line total H3K4me3 (green), in-line total H3K27me3 (red) and bivalent K4-K27 (purple) and K27-K4 (blue) reChIP data tracks are shown. Dppa2/4-dependent promoters (lose bivalency when DPPA2/4 absent) are denoted by orange bars. Stable promoters are denoted by blue bars. **C** Scatterplots showing enrichment (log_2_ CPM/bp) for K4-K27 (top) and K27-K4 (bottom) reChIPs between wild type (x-axis) and Dppa2/4 double knockout (DKO) (y-axis) across all bivalent peaks (grey). Highlighted are those differentially enriched in the K4-K27 (purple), K27-K4 (blue) or both (orange) reChIP datasets. **D** box plot showing normalised enrichment (CPM/bp) of previously annotated Dppa2/4-dependent gene promoters (light orange) and novel Dppa2/4-dependent gene promoters (dark orange) across the different datasets and clones. As a comparison a subset of stable Dppa2/4-independent gene promoters (bivalent promoters that do not change) are shown (blue). **E** Enrichment heatmaps showing normalised enrichment of previously annotated Dppa2/4-dependent genes (top, light orange) and novel DPPA2/4-dependent genes (middle, dark orange) across the different datasets averaging across clones. As a comparison a subset of Dppa2/4-independent genes (bivalent promoters that do not change) are shown (bottom, blue). **F** Log_2_ RPKM expression levels of original (light orange), novel (dark orange) Dppa2/4-dependent genes and high confidence but not differentially enriched (blue) genes across the different datasets between wild type (WT) and DPPA2/4 double knockout (DKO) cells. As a comparison the bottom 20% (light grey) and top 20% (dark grey) expressed genes are shown. **G** log_2_ normalised expression levels of previously annotated (light orange, top), novel (dark orange, middle) Dppa2/4-dependent genes compared to stable bivalent genes (blue, bottom) during 9 days of mouse embryoid body differentiation in wild type cells (left) and DPPA2/4 double knockout cells (right). Each gene has been normalized separately across the time series to aid visualisation of expression patterns, genes are grouped using correlation based clustering

Lastly, we confirmed the sensitivity of our method to detect changes in bivalent chromatin by profiling mouse embryonic stem cells deficient for the epigenetic priming factors DPPA2 and DPPA4 [7]. We and others recently reported that DPPA2/4 are required to maintain bivalent chromatin at a subset of bivalent genes which were termed Dppa2/4-dependent [6, 7] (Fig. 5A). To test the dynamic sensitivity of our method, we profiled two wild-type (WT) and two DPPA2/4 double knockout (DKO) clones using our refined method. Our reChIP datasets recapitulated previous observations where total H3K4me3 and total H3K27me3 signals were lost at Dppa2/4-dependent bivalent genes yet retained at Dppa2/4-independent (stable) genes in the DPPA2/4 knockout cells (Fig. 5B). Importantly, this was also observed in the both bivalent K4-K27 and K27-K4 reChIP directions, highlighting the ability of our improved method to detect dynamics of bivalent chromatin between different conditions.

Given the improved sensitivity of our method we next sought to determine whether there may be more widespread changes in chromatin bivalency in DPPA2/4 knockout ESCs compared to what had been previously reported [6, 7]. Firstly, we called peaks for the reChIP samples. This revealed 9,225 peaks that were bivalently marked in either wild-type and/or DPPA2/4 DKO cells in both reChIP directions (Additional file 2: Figure S4A) of which 5,713 were high-confidence in either WT and/or Dppa2/4 DKO cells. To determine if any peaks were gained or lost specifically in Dppa2/4 DKO cells, we performed differential enrichment test using EdgeR (Additional file 3: Table S5, see methods). There were 566 differentially enriched bivalent regions in both the K27-K4 and K4-K27 reChIP datasets (Fig. 5C). Consistent with previous results [6, 7], all the differentially enriched peaks were downregulated or absent in the DPPA2/4 DKO cells and there were no upregulated peaks (Fig. 5C). This included promoters previously described as Dppa2/4-dependent [7], but also novel Dppa2/4-dependent bivalent regions detected using the increased sensitivity of our method.

Promoter-centric analysis revealed differential enrichment of both bivalent K4-K27 and K27-K4 at 266 gene promoters of which 238 were high confidence (Additional file 2: Figure S4A). When compared to those previously reported [7], we confirmed 141 Dppa2/4 dependent promoters and identified an additional 125 novel Dppa2/4-dependent bivalent promoters. The newly identified (novel) Dppa2/4-dependent promoters had similar levels of enrichment of total H3K4me3, total H3K27me3 and K4-K27 and K27-K4 reChIPs compared to previously known (original) Dppa2/4-dependent promoters (Fig. 5D, E). Both original and novel Dppa2/4-dependent bivalent promoters were similarly expressed in undifferentiated ESCs (Fig. 5F), at lower levels than stable bivalent promoters which did not lose bivalency. Moreover, similar to original Dppa2/4-dependent promoters [7], the novel Dppa2/4-dependent promoters also failed to be upregulated upon embryonic stem cell differentiation while stable bivalent promoters remained dynamically expressed (Fig. 5G). In summary, this proof-of-principle experiment supports the ability of our method to detect dynamic changes in bivalent chromatin landscapes with high sensitivity and resolution.

## Discussion

Here, we present a refined low-input sequential ChIP-reChIP method to robustly and accurately map bivalent chromatin genome-wide. Compared to previously published methods and datasets our approach has several advantages. Firstly, the method requires a substantially reduced input number of cells with just 2 million cells sufficient to generate high quality H3K4me3-H3K27me3 and H3K27me3-H3K4me3 reChIP datasets along with in-line total H3K4me3, total H3K27me3 and IgG–IgG controls. This is a dramatic improvement from the typically 10 million cells or more needed per dataset in other methods [13, 14, 22], and will facilitate the investigation of these domains in samples where cell numbers are limiting. Next, the data generated has very clear peak enrichments with low background enabling standard peak-calling and bioinformatic pipelines to be used to call and classify bivalent regions. While we optimized this method in mouse embryonic stem cells, we envisage its widespread applicability in many different cell lines and tissues.

In this study, we reveal 8,789 bivalent regions corresponding to 5,104 promoters in mouse embryonic stem cells. Consistent with our current understanding of bivalent chromatin, most bivalent promoters contained CpG islands and were associated with genes involved in developmental processes. Of the non-promoter bivalent peaks, we observed many were enriched at candidate enhancer regions, suggesting a potential regulatory role for bivalent chromatin at these elements. However, due to the reported cross-reactivity of the H3K4me3 antibody used in this study with H3K4me2 [24], the enhancer-associated bivalent chromatin regions may reflect H3K4me2-H3K27me3 co-occurrence as opposed to the classic promoter-associated H3K4me3-H3K27me3 combination.

A key step in all chromatin immunoprecipitation experiments is generating high quality mononucleosomes. The method presented here uses MNase digestion, however, we have successfully performed bivalent reChIP experiments from similar number of cells using sonication to shear chromatin with very similar results (Additional file 2: Figure S1A, B). Importantly MNase/sonication conditions must be optimized for each cell type to ensure predominantly mononucleosome distribution. Over-digested chromatin may not perform well in immunoprecipitation reactions, while under-digested chromatin will confound downstream analysis as it decreases the genomic resolution that can be analysed. In our protocol, we implemented a pre-clearing step and found that this drastically improved the signal-to-noise in our experiments. By pre-incubating chromatin with dynabeads, non-specific binding of chromatin fragments to the beads is reduced, removing background and facilitating lower input amounts. Our protocol uses many wash rounds following the immunoprecipitation reactions. We found these to be critical to achieve low background levels. Lastly, we also tested different elution conditions and found that while both SDS-based and peptide-elution approaches behaved similarly, peptide competition elution had higher background levels at long incubation times. Either method could be used in reChIP protocols, however, due to cost and availability we opted for SDS-based elution followed by buffer exchange and chromatin concentration prior to the second immunoprecipitation reaction.

Controls are an important part of any experimental design. The IgG–IgG reChIP control provides an estimation of the level of background non-specific binding. When possible, we recommend running a diagnostic qPCR for known bivalent regions and controls prior to library preparation and sequencing. If the IgG–IgG reChIP pulldown amounts are high by qPCR analysis this often indicates the reChIP experiment has not performed well. The IgG–IgG can also be used as normalisation for peak calling in addition to or instead of input samples. Perhaps the most critical control is to perform the reChIP experiment in both orientations (H3K4me3 followed by H3K27me3 and vice versa). Our analysis revealed several thousand peaks that are detected in one but not the other reChIP dataset, likely due to the first immunoprecipitation signal carrying through non-specifically from the first immunoprecipitation into the second. This is a common caveat in sequential ChIP experiments and extremely hard to completely eliminate. Therefore, to control for this, any reChIP experiment should always be performed in both orientations to be sure that the detected peaks are indeed due to the presence of both marks on the chromatin.

We also explored other controls that have been used by other studies. One commonly used control is to perform the first immunoprecipitation using H3K4me3 or H3K27me3 and then follow this with a second immunoprecipitation using IgG [22]. The rationale behind this is that IgG is non-specific and so there should be no final overall enrichment. However, in our experience, we found that H3K4me3-IgG or H3K27me3-IgG reChIPs mirrored the first immunoprecipitation (Fig. 2F, Additional file 2: Figure S1H). IgG immunoprecipitation will randomly sample from the pool of chromatin and so if performed as the second immunoprecipitation, this will subsample the already enriched H3K4me3 or H3K27me3 pool of chromatin (Fig. 2G). Consequently, we have not found this to be a useful control in our experiments or analyses. Instead, to control for signal carry-over from the first IP into the second, we treated cells with the Ezh2 inhibitor Tazemetostat which results in global loss of H3K27me3 levels, and found that this depleted the reChIP signal in both directions at bivalent regions (Fig. 2H), indicating we are indeed enriching for *bone fide* bivalent chromatin in our assay.

When assessing bivalent chromatin, many studies have performed in silico merges of independently derived H3K4me3 and H3K27me3 datasets. Theoretically this is unable to distinguish between bone-fide bivalent chromatin from allelic or sample heterogeneity. In our data, 26.3% of bivalent regions identified this way are false-positives and were not captured by sequential reChIP in both directions. This is similar to false-positive rates described of 25% in mouse ESCs [14] and 14% in human T-cells [13]. Not only does the in silico overlap approach have a high false-positive rate, it also misses bivalent regions. We also reveal thousands of additional bivalent regions that have a peak in both reChIP orientations but not in in silico estimations. These false-negative bivalent regions may be bivalent in only a subset of cells or alleles and thus only detectable through the sequential enrichment possible through reChIP protocols. Thus, our results demonstrate the importance of performing reChIP to profile heterogeneous or complex samples containing multiple cell types and states. Whether this is the case in other cell types remains unknown.

## Conclusions

Our refined sequential reChIP method provides a useful resource for the wider epigenomics and chromatin biology fields. The optimized protocol accurately and robustly detects bivalent regions in mouse embryonic stem cells from as little as 2 million cells. Consistent with current models, the bivalent regions occur predominately at CpG-rich promoters that are dynamically regulated during differentiation. Lastly, our analysis of DPPA2/4 knockout cells confirms the ability of our method to detect changes in the bivalent chromatin landscape. Our protocol uses readily available reagents and equipment found in most molecular biology laboratories and can be adapted to profile this unique form of epigenetic plasticity in any cellular context with the confidence that any conclusions are free from potential confounding effects of cellular heterogeneity. This method will facilitate accurate profiling of the dynamics of bivalent chromatin in other contexts, greatly improving our understanding of this unique form of epigenetic plasticity.

## Methods

### Cell culture

Mouse embryonic stem cells were cultured on feeder-free gelatinised plates at 37 ℃, 5% CO_2_ using standard serum/LIF conditions (high-glucose DMEM supplemented with 15% fetal bovine serum, 1 × GlutaMax, 1 × penicillin, 1 × streptomycin, 0.1mM nonessential amino acids, 50mM beta-mercaptoethanol and LIF (made in house in HEK293 cells and titrated for optimal ESC growth)). Cells were regularly tested for mycoplasma contamination using the Mycoplasma PCR Detection Kit (abcam ab289834). E14 mouse embryonic stem cells were a gift from W. Reik’s laboratory. Wild type and DPPA2/4 double knockout clones were generated in [7, 29] and cultured as above. To deplete global H3K27me3 levels, cells were treated with 10μM Ezh2 inhibitor Tazemetostat (GSK126) for 7 days, which has been previously shown to deplete H3K27me3 in mESCs [26]. Cells were not authenticated. Cells were cultured at least 2 passages from thawing prior to chromatin collection. Biological replicates were collected from different passages on separate days.

### Cell collection and fixation

Cells were seeded on multiple plates and grown to near-confluency. At time of harvest one plate was used to determine cell concentration. Cells on remaining plates were washed with PBS and fixed with 1% methanol-free formaldehyde (Thermo Scientific 28908) in DMEM at room temperature for 8 min, quenched with 0.125M glycine and scraped off cell culture dishes. Cell slurry was washed with ice-cold PBS, resuspended in PBS/EDTA, aliquoted to 2 × 10^6^ cells per vial, spun down and snap frozen on dry ice for storage at –80°C. Cell pellets were used within 6 months of collection.

## Sequential chromatin immunoprecipitation

A detailed protocol accompanies this paper (Additional file 1). Pellets of 2 × 10^6^ cells were lysed with 100μl NP buffer (10mM TrisHCl pH7.4, 1M sorbitol, 50mM NaCl, 5mM MgCl_2_, 0.075% IGEPAL) freshly supplemented with 0.385mM beta-mercaptoethanol (Gibco 21985–023) and 1.8mM spermidine (Sigma 05292) on ice. Chromatin was digested using 2.4μl per sample of MNase (NEB) for 37 ℃ for 15 min with gentle shaking at 600rpm. Reactions were stopped with 26.4μl STOP buffer (50mM EDTA, 0.5% TritonX-100, 0.5% sodium deoxycholate), incubated on ice for > 5 min, vortexed and sample diluted to 580μl in ChIP buffer (20mM TrisHCl pH8.0, 2mM EDTA, 150mM NaCl, 0.5% Triton X-100) containing protease inhibitor cocktail (cOmplete EDTA-free Protease Inhibitor Cocktail, Roche). For sonication comparisons, pellets of of 2 × 10^6^ cells were lysed with 100μl NP buffer, diluted to 580μl in ChIP buffer and sonicated using the Covaris ME220 (water temperature 4°C, Peak power 75W, Duty factor 75%, cycles per burst 1000, Run time 20 min). Chromatin digests/sonications were regularly confirmed through gel electrophoresis to ensure predominantly mononucleosomal fragments.

Chromatin was precleared by adding 20μl prewashed Protein A dynabeads (Invitrogen 10002D) and incubating at 4°C on rotator for > 2 h. 5% of the sample was set aside as input, the remaining chromatin was divided amongst separate tubes containing protein A dynabeads pre-incubated with either 2μl anti-H3K4me3 (Millipore 07–473), 10μl anti-H3K27me3 (CST 9733) or 1μg IgG (Invitrogen) antibodies. Cell dilution experiments in Additional file 2: Fig. S1C were performed using anti-H3K27me3 antibody from Active Motif (91167). We note that the Millipore H3K4me3 antibody used in this study has been reported to also detect H3K4me2 [24], and so it is possible we are also deteting H3K4me2-H3K27me3 bivalent nucleosomes. The first immunoprecipitation was performed overnight at 4 ℃ with rotation. Antibody-chromatin complexes were washed 3 × in low salt buffer (20mM TrisHCl pH8.0, 2mM EDTA, 150mM NaCl, 1% Triton X-100, 0.1% SDS), 3 × in high-salt buffer (20mM TrisHCl pH8.0, 2mM EDTA, 500mM NaCl, 1% Triton X-100, 0.1% SDS), 2 × in LiCl buffer (0.35M LiCl, 1% IGEPAL, 1% sodium deoxycholate, 1mM EDTA, 10mM Tris–HCl pH7.5) and 2 × in TE on ice. For single-ChIPs complexes were eluted in 20-50μl elution buffer (10mM TrisHCl pH8.0, 1mM EDTA, 1% SDS) for 65 ℃ for 2.5 hours to overnight to reverse cross-links, treated with RNAseA (NEB) for 30 min at 37 ℃, proteinase K (NEB) for 1 hour at 37 ℃, and purified using NEB DNA purification columns. For sequential ChIPs, complexes were eluted in 100μl elution buffer containing fresh protease inhibitor cocktails for 30 min at 37 ℃ with shaking. 10% sample was set aside as total in-line control ChIPs. To dilute SDS volume was increased to 300μl with ChIP buffer containing protease inhibitor cocktails and purified using Amicon Ultra-0.5ml 3KDa filter columns (Millipore) according to manufacturers’ instructions, recovering approximately 50μl chromatin per IP reaction. The second immunoprecipitation was performed using the alternate antibody or IgG control overnight at 4 ℃ with rotation and chromatin washed and eluted as previously. In-line control, input and ChIP samples were heated at 65 ℃ for 2.5 h to reverse cross-links, treated with RNAseA (NEB) for 30 min at 37 ℃, proteinase K (NEB) for 1 h at 37 ℃, and purified using Ampure beads (Beckman Coulter) at a 1:1.8 ratio.

### Peptide elution experiments

Peptide elution experiments were performed by resuspending washed dynabead-antibody-chromatin complexes in 200μl peptide elution buffer (50mM Tris–HCl pH8.0, 5mM EDTA, 100mM NaCl, 0.5% sodium deoxycholate, 0.1% SDS) supplemented with protease inhibitors containing 10μg/ml H3K4me3 (abcam ab1342) or H3K27me3 peptides (abcam ab1782) on rotator at 4 ℃ for 3 h or overnight. For the IgG control sample 10μg/ml of a 1:1 mix of H3K4me3 and H3K27me3 peptides was used.

### qPCR analysis

qPCR analysis of purified ChIP DNA was performed in technical duplicate for each primer pair using 2 × SYBR mastermix (Applied Biosystems Cat#4385612) according to manufacturer’s instructions in a 6-10μl reaction using the primer sequences as below.

H3K4me3-only controls.

Klf4_forward GAAAGTCCTGCCACGGGAA.

Klf4_reverse CTGGATGAGTCACGCGGATAA.

Dppa2_forward GCCAAACACAGACTACGCTA.

Dppa2_reverse AACCTACACTATTTTCGCCAGGAT.

Dppa4_forward TTCTCAAGATGGAGACTGCTGG.

Dppa4_reverse TGGCTATACTCAAAAATGAGGGGC.

Gapdh1_forward AGTGTGCACCAAGGACATCCAG.

Gapdh1_reverse CCCATTTTACTCGGGAAGCAG.

H3K27me3 only controls.

Gm6116_forward GCGGTGAGTACTCTGCTCAA.

Gm6116_reverse CCATCCAGTACTGTGGGCTC.

K27me_R1_forward TGCCTGCAATTCGTCCTCTT.

K27me_R1_reverse ACGAAGCAGCCGTGTAAGAA.

Meis2_forward TGCCATTACTTGAGACAGAGCACCAC.

Meis2_reverse GAGGGAACATGAGTGGTC.

Bivalent regions.

Csf1_forward GAGCACCGAGGCAAACTTTC.

Csf1_reverse GAGCCAGGGTGATTTCCCAT.

Lmo1_forward AAGCGGGCTCTAATTACCCG.

Lmo1_reverse CTGCGAAGTGCTTCACTCCT.

Pou4f1_forward CAAAGTGAGGCTGCTTGCTG.

Pou4f1_reverse GCGGACTTTGCGAGTGTTTT.

Sox6_forward CGATACAGAAGCGCAGGCTA.

Sox6_reverse AGGGGCCCTTGTAGATGGAT.

Colgalt2_forward ATGAGGACGGAGAGGAAACG.

Colgalt2_reverse CATCCTGCTCTTGGGGTAGT.

Pfsd4a_forward CTATCCCTCTCCAGTGCCAG.

Pfsd4a_reverse GAGTGATCCGTGGCAATTCG.

Rnf149_forward GAAGAGACAATGCGAGCCTG.

Rnf149_reverse TCTACAGATTCCCGAACCCG.

Mcmdc2_forward GCAACATAGCCAGGTCGAAG.

Mcmdc2_reverse CCCAAACACCTCAGGACTCA.

Tlx1_forward CATACACCTCGGCCTTCCTC.

Tlx1_reverse GCACGGAGCTCAGGGAATAA.

### Library preparation

Sample DNA concentrations were quantified using Qubit and libraries prepared using NEBNext Ultra II DNA library preparation kit (NEB) according to the manufacturer’s instructions with the following modifications. To achieve optimal final DNA concentrations, samples were re-quantified on the Qubit 3.0 following PCR amplification and an additional 3 cycles (if concentration close to 20ng/μl) or 5 cycles (if concentration <  < 20ng/μl) performed if needed to obtain the ideal final library concentration of 20-100ng/μl. A maximum of 20 cycles was used for any sample. Note that due to the low amounts of DNA obtained from the protocol, concentration measurements prior to amplification typically occur at the lower limit of detection and even if zero values are obtained, libraries can often still be generated. Libraries were purified using NEBNext sample purification beads and checked using Agilent Tapestation 2200 or 4150 on a high-sensitivity tape (HSD1000) aiming for a final library with dominant peak size of 270bp. Libraries were pooled and sequenced using the Illumina NextSeq500 platform with a target read depth of 20 million SE75bp reads per sample except for replicate 2 which was intentionally sequenced deeper.

### Data pre-processing

Single-end reads in fastqs were trimmed and filtered for quality (phred33 score > 20) and length (> 20bp) using TrimGalore (https://github.com/FelixKrueger/TrimGalore) v0.6.6 in single-end mode. Trimmed and quality filtered reads were then aligned to the mm10 mouse genome (GRCm38.p6) using bwa-mem [30] (bwa v0.7.13) with default parameters. Alignments were then converted to the bam format and indexed using samtools v1.9. Duplicate alignments were then marked with using *MarkDuplicates* (picard v2.6.0, https://broadinstitute.github.io/picard/) and re-indexed with samtools [31]. bigWig files containing CPM/bp normalised coverage values for each sample were derived from duplicate marked bam files using *bamCoverage* (DeepTools v3.5.0 [32]) whilst excluding ENCODE blacklisted genomic regions [33] for the mm10 genome (v2).

### Software and datasets

Downstream data analyses were conducted using R (v4.1.2) or SeqMonk (v1.48.1) (http://www.bioinformatics.babraham.ac.uk/projects/seqmonk). In-line total H3K4me3, in-line total H3K27me3, IgG–IgG, bivalent K4-K27 and bivalent K27-K4 datasets for E14 ESCs, Dppa2/4 WT clones and Dppa2/4 DKO clones were generated in this study. Single-ChIP-seq data from 10 million cells of H3K4me3, H3K27me3 and input controls were obtained from [7] (GSE135841). Previously published single H3K4me3 and H3K27me3 ChIP, and H3K4me3-IgG, H3K27me3-IgG, H3K4me3-H3K27me3 and H3K27me3-H3K4me3 reChIP datasets were reanalyzed from [14] (GSE99530). Gene expression data were obtained from [7] (GSE135841).

### ChIP and reChIP data analysis

Peak calling for in-line total H3K4me3, total H3K27me3 and K4-K27 and K27-K4 reChIP datasets were performed separately for the two biological replicates using EPIC2 (v0.0.52) [34] (–bin-size 100 –gaps-allowed 1 –fragment-size 147 – false-discovery-rate-cutoff 0.05) using IgG–IgG as control. EPIC2 is an ultrafast implementation of the original SICER peak calling algorithm which performs well for diffuse histone marks. For total H3K4me3 and total H3K27me3 data derived from 10 million cells, input DNA was used in peak calling. Peaks were filtered (log_2_FC > 2 and FDR < 0.05) and regions overlapping blacklisted regions from the mm10 ENCODE blacklist [33] were excluded. To derive commonly enriched regions, intersections between replicates were performed using the GenomicRanges R package (*intersect, subsetByOverlaps*) (v1.46.1). To derive in silico bivalent regions total H3K4me3 and total H3K27me3 peak sets were intersected once more. Similarly, K4-K27 and K27-K4 reChIP peaks were also intersected to derive reciprocally bivalent regions. For differential expression analyses, a consensus peak set was first derived using the GenomicRanges R package (*Reduce* and *union*). Reads were counted at consensus peaks using the csaw R package (*regionCounts*) (v1.28.0) [35]. Normalisation factors for each sample were pre-computed by binning the genome into 10kb windows, generating counts for each 10kb window and performing trimmed mean of M-values (TMM) normalisation using csaw (*normFactors*) to adjust for compositional biases. Differential enrichment was then performed using EdgeR (*estimateDisp* > *glmQLFit(robust* = *TRUE)* > *glmQLFTest*) (v3.36.0) in which double knockout clones (DKO) were contrasted with wild-type clones (WT) for each reChIP (K4-K27, K27-K4). Benjamini–Hochberg p-value corrections for multiple testing were applied to these contrasts. Significant differential enrichment was defined as a region that had |log_2_FC|> 1 and FDR < 0.05.

Peaks were annotated using HOMER (*annotatePeaks.pl*) (v4.11) which by default denotes promoter-TSS regions as 1kb upstream and 100bp downstream of transcription start sites (TSS). Promoter-TSS regions were re-defined as the region spanning 1.5kb upstream and 1.5kb downstream of transcriptional start sites (TSS). For hierarchically classifying promoter-TSSs, we assigned each unique promoter-TSS to a singular peak based on their classification (HC > K4b > K27b > LC). CpG island and ENCODE cis-regulatory element intersections were performed using the GenomicRanges R package (*findOverlaps*) and were obtained for the mm10 genome using the UCSC table browser [36].

For each peak set, the fraction of reads in peaks (FRiP) was calculated by first counting the reads in each bam at each peak with the csaw R package (v1.28.0) and then dividing the sum of these counts by the total number of reads in the library were performed using samtools (*idxstats*) (v1.9) and sambaba (*view –subsampling-seed 1*) (v0.6.7).

In SeqMonk (v1.48.1), aligned read (bam) files were imported using standard parameters (no deduplication, MAPQ > 20, primary alignments only), extending reads by 200 bp. Normalised read densities within peaks were calculated as log_2_-transformed read counts in peaks corrected for library size (counts per million reads) and peak length (per bp) yielding CPM/bp. Box plots represent median (horizontal line), 25th–75th interquartile range (box) and 2 × the 25th-75th interquartile range (whiskers) with outliers shown as dots. Scatterplots compare log_2_CPM/bp values for either peaks called using EPIC2 (Figs. 3C, 4D, E, 5C) or 200bp sliding window bins across the genome (Additional file 2: Figure S1H). Relative distribution probe trend plots weight each probe equally in the final average profile at 1bp resolution. Per-probe normalized hierarchical clustered heatmaps are ordered using correlation based clustering to group probes (genes) with similar shaped quantitation profiles. Values are normalised across the set of samples (median value for each gene is subtracted from the actual value for that gene in each of the samples) to enable easier comparison.

### Gene expression analysis

Data were trimmed with Trim Galore (v0.4.4, default parameters) and mapped using HiSat2 v2.1.0 to the mouse GRCm38 genome assembly. RNA-sequencing analysis was performed using SeqMonk software using inbuilt RNA-sequencing quantification pipeline. Expression values represent log_2_ transformed quantification of merged transcripts counting opposing-strand reads over exons. Gene expression heatmaps are normalized for each transcript independently by subtracting the median value for that transcript across all samples from each sample value. Bean plots represent smoothed density of all points over the bandwidth window corresponding to 5% of the total quantitation range displayed in the plot.

### Genomic enrichment heatmaps and trackplots

To generate genomic enrichment heatmaps and trackplots bigWigs containing CPM/bp normalised read densities were imported to R using the rtracklayer package (*import.bw*) (v1.54.0). For heatmaps, each peak region was first extended to 5kb upstream and downstream and then split into 100 equally sized bins using the GenomicRanges R package (*resize*) (v1.46.1) [37]. The average CPM/bp was calculated for each bin for each ChIP using the EnrichedHeatmap R package (*normalizeToMatrix*) (v1.24.0) [38]. Bins with values surpassing the 99th percentile of all bins within each ChIP were masked (i.e., assigned the 99th percentile value) to eliminate extreme outliers from affecting colour scales. Each bin was then scaled relative to the highest value (so values range between 0 and 1 and represent the relative enrichment of signal across all regions). Enriched heatmaps were then plotted using the same package (*EnrichedHeatmap*), with the average bin value plotted as continuous curves atop each heatmap. Genomic track plots were plotted using the rtracklayer (v1.54.0) [39] and Gviz R packages (v1.38.4) [40]. CpG island annotations for the mm10 genome were retrieved from the UCSC table browser [41].

### Gene ontology

The enrichment of gene ontologies across subclasses of genes with bivalent promoters (HC, K4b, K27b, LC) were determined using the clusterProfiler R package (v4.2.2) [42]. Gene symbols were first converted to entrez IDs using the biomaRt R package (v2.50.3) [43] and were input alongside a background list of all expressed genes to clusterProfiler (*compareCluster)* against the Gene Ontology (GO) Biological Processes (BP) database [44]. Significantly enriched GO terms were those with a Benjamini–Hochberg (BH) corrected p-value < 0.05, had at least 10 genes present in the pathway and a gene ratio (genes in subclass/genes in pathway) > 0.01. Representative pathways were plotted using the ggplot2 R package (v3.3.5) [45].

### Motif analysis

Enrichments for transcription factor binding motifs in peak subclasses were calculated using the monaLisa R package (v1.0.0). Position weight matrices for transcription factor binding sites in vertebrates were retrieved from the JASPAR2020 database [46]. Binned motif enrichment for peak subclasses (HC, K4b, K27b, LC) was then conducted in monaLisa [47] (*calcBinnedMotifEnrR*) while including randomised sequences modelled off the width and re-sampled based on the GC-content of all bivalent peaks (made with the regioneR R package (*createRandomRegions*) (v1.26.1) [48] and nullranges R package (*matchRanges*) [49] providing GC-content as a covariate) (v1.0.1) as the background. Significant enrichments were those with a BH-adjusted p-value < 0.05 and log_2_-fold enrichment over random sequences > 1. Motif heatmaps were also plotted using monaLisa (*plotMotifHeatmaps*).

### CG content

CG content was determined for peak subclasses (HC, K4b, K27b, LC) as well as the same random control sequences above using the Biostrings R package (*oligonucleotideFrequency*) (v2.62.0) [50] by first calculating all oligonucleotide frequencies and then by summing C and G frequencies. All dinucleotide frequencies were calculated using monaLisa (*plotBinDiagnostics*) and then GC/CG dinucleotide frequencies were summed. These data were plotted using ggplot2, and the significance of comparisons were determined using pairwise t-tests followed by BH-adjustments of p-values to account for multiple comparisons.

### Chromatin state discovery

Bam files were first converted to the bed format using bedtools (*bamtobed*) (v2.27.1) [51]. bed files were then partitioned into 200bp bins and then binarized for the determination of bin-specific enrichments (providing replicate in-line ChIPs or reChIPs and using IgG–IgG as the control) using ChromHMM (*BinarizeBed*) (v1.24) [52]. Hidden Markov Models were then used to discover chromatin states across these genomic bins using ChromHMM (*LearnModel*) using a 5-state model. Segment bed files containing chromatin state annotations were then overlapped with our bivalent peak annotations (HC, K4b, K27b, LC), where each peak was then re-assigned to the chromatin state with the highest degree of overlap using the GenomicRanges R package (*findOverlaps* and *pintersect*) (v1.46.1) [37]. Heatmaps containing emission probabilities, transition probabilities, TSS enrichments and annotation overlaps from the ChromHMM model were then plotted using the ComplexHeatmap R package (v2.10.0) [53].

### Software

Plots were generated using SeqMonk software (v1.48.1) or R (v4.1.2/RStudio v2022.02.0 + 443) and edited in Inkscape. Schematic figures were made with BioRender.com with publishing licence agreement numbers *RM25UDOLG3, UQ25UDOE0U* and *MC25UDOPHO.*

### Supplementary Information

**Additional file 1: **Detailed step-by-step protocol for ChIP-reChIP.

**Additional file 2: Figure S1.** (A) Agarose DNA gel showing chromatin fragmented with MNase versus sonication (B) ChIP-qPCR for single (top) and reChIP (bottom) experiments using IgG (Invitrogen, black), H3K4me3 (CST 9751, green) and H3K27me3 (CST 9733, red) antibodies on sonicated chromatin. An active H3K4me3 region (Gapdh1), inactive H3K27me3 region (Meis2) and 6 bivalent regions are assayed, four of which are novel to this study. (C) Single ChIP-qPCR analysis using IgG (Invitrogen, left), H3K4me3 (Millipore 07-473, middle) and H3K27me3 (Active Motif 91167, right) antibodies with variable number of cells ranging from 1 million (1M), through to 2,000 (2K). Five primer sets were used including three H3K4me3 enriched regions (green) and two H3K27me3 enriched regions (red). Note that below 50K the background increases and specificity of enrichment is no longer detected. For this experiment a different H3K27me3 antibody was used to the sequenced reChIP datasets. (D, E) downsampling analysis for K4-K27 and K27-K4 datasets using the higher coverage replicate 2. Peaks were called separately for each downsampled dataset and classified according to Figure 3A. (D) Total peaks, (E) promoter peaks. (F, G) Downsampling analysis of independent total H3K4me3 and total H3K27me3 from (GSE135841) (7) showing *in silico *bivalent predictions atall peaks (G) and promoter peaks (H) at different simulated read depths. (H) Pseudocolour density scatterplot showing log_2_ CPM/bp enrichment at combined H3K4me3 and H3K4me3-IgG peaks for total H3K4me3 single ChIP (x-axis) compared to H3K4me3-IgG reChIP (y-axis). R=0.906. Data reanalysed from Mas et al. 2018 (14). **Figure S2.** (A) Schematic showing peak calling strategy for *in silico* bivalent peak prediction (B-F) Genome browser views of (B) H3K4me3-only, (C) high-confidence, (D) K4-biased, (E) K27-biased, and (F) low confidence genes showing H3K4me3 (green), total H3K27me3 (red) and K4-K27 (purple) and K27-K4 (blue) reChIP datasets. Height of peak represents CPM/bp. E-M represents data from independent 10 million cell total H3K4me3 and total H3K27me3 (GSE135841) (7). R1 and R2 are two independent biological replicates from this study. (G) number of bivalent promoters overlapping (dark grey) or not-overlapping (light grey) CpG islands for the four different classes. Genome-wide 51.97% promoters overlap a CpG island using these criteria (H) Overlap of non-promoter bivalent regions with candidate cis-regulatory regions (cCREs) from ENCODE for the four different classes. **Figure S3.** (A) Classification strategy for calling bivalent promoters in data from Mas et al. 2018 (14). Note only one replicate of the reChIP datasets were generated in this study. Numbers denote number of peaks/promoters at each step. (B) Enrichment of bivalent promoters shared between this study and Mas et al. 2018 (14) (top, n=3593) or unique to our study (bottom, n=1511) in Mas et al. 2018 (14) datasets. (C) log_2_ fold change in gene expression levels for pluripotency genes (top) and a random set of expressed non-bivalent genes (bottom) across 9 days of embryoid body differentiation. Each gene has been normalised separately across the time series. Top and bottom groups of genes are on different scales. Gene expression data reanalysed from (GSE135841). **Figure S4.** (A) Classification strategy for calling differential bivalent promoters between wild type (WT) and Dppa2/4 double knockout (DKO) clones. Numbers denote number of peaks/promoters at each step.

**Additional file 3: Table S1.** library statistics, list of all bivalent peaks and list of peaks called in each replicate for total H3K4me3, total H3K27me3, bivalent K4-K27 and K27-K4 reChIP datasets generated in this study (E14 rep1 and rep2), independently generated (GSE135841) and Dppa2/4 WT (clones 57 and 58) and double knockout (clones 43 and 53) samples. In the list of all bivalent peaks (tab 2), peaks are classified as high-confidence (hc), K4-biased (k4b), K27-biased (K27b), or low confidence (lc) using either in-line total ChIPs (column E) or independent total ChIPs (column F).** Table S2.** motif analysis of bivalent peaks using monaLisa along with associated statistics. **Table S3.** list of bivalent genes classified as high confidence, K4-biased, K27-biased and low confidence along with log_2_ CPM/bp enrichment scores for independent total H3K4me3 and H3K27me3, IgG-IgG, in-line total H3K4me3 and H3K27me3 ChIP and K4-K27 and K27-K4 bivalent reChIP datasets. **Table S4.** Gene ontology enrichment of different bivalent gene classifications (first column) together with associated statistics and list of associated genes. **Table S5.** list of EDGER differentially enriched peaks for bivalent K4-K27 (tab1) and K27-K4 (tab2) reChIP between Dppa2/4 WT and DKO clones. Consensus differentially enriched peak list is shown in tab 3 along with HOMER classification of peak, enrichment statistics and (for promoters) whether they were previously classified as differentially enriched.

## Data Availability

The datasets generated during the current study have been deposited in the short read archives (SRA) and gene expression omnibus (GEO) under the accession GSE242686. Gene expression data and previous ChIP-seq data was obtained from [7] (GSE135841) and [14] (GSE99530).
