# Supplementary material for: A low-input high resolution sequential chromatin immunoprecipitation method captures genome-wide dynamics of bivalent chromatin
Source: Epigenetics Chromatin. 2024 Feb 10;17:3. doi: 10.1186/s13072-024-00527-9 (PMC10858499; doi:10.1186/s13072-024-00527-9)

Seneviratne, Ho et al. Supplemental Figure 2

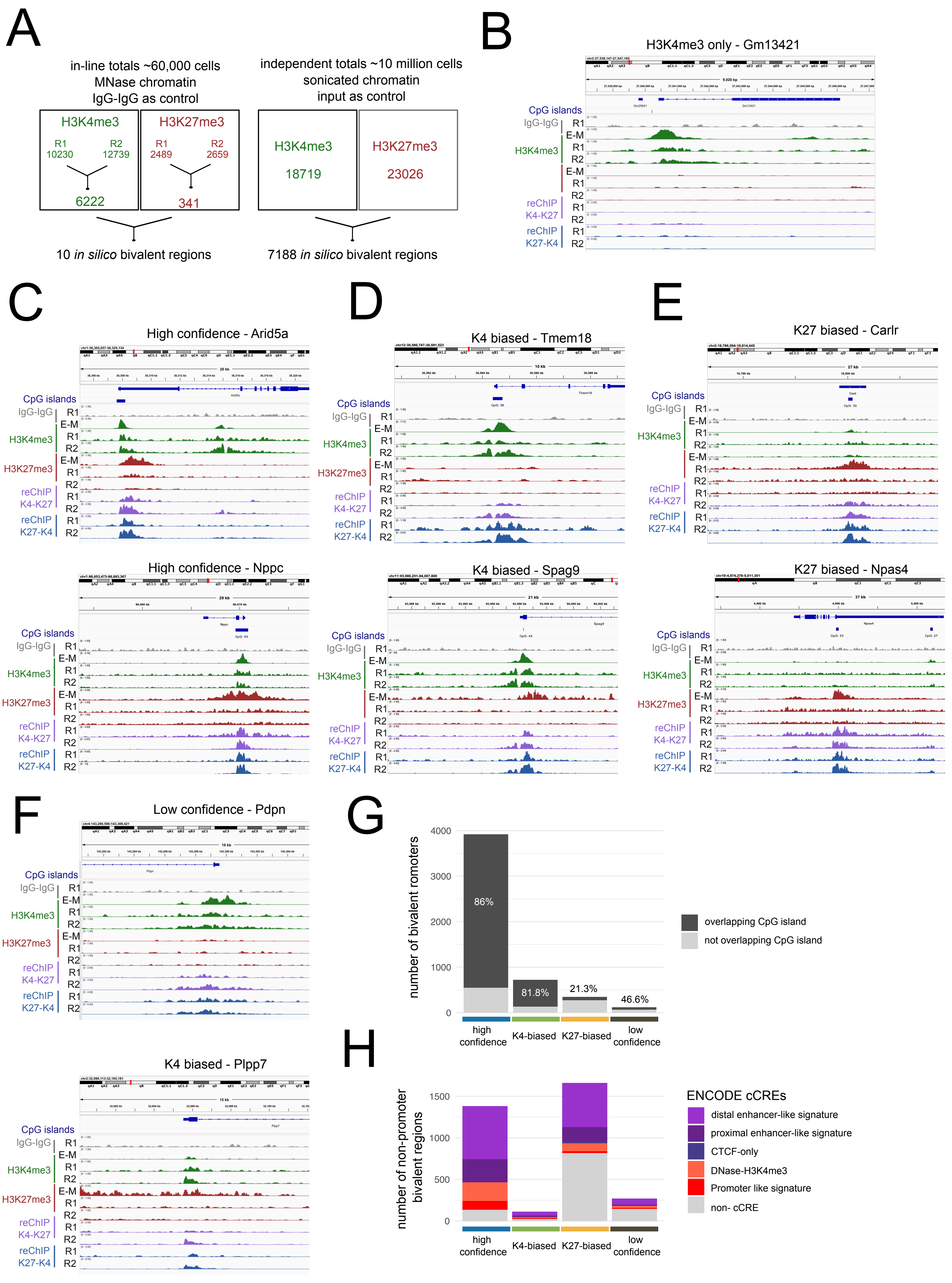

Supplement: Supplementary file 2 — Additional file 2: Figure S1. (A) Agarose DNA gel showing chromatin fragmented with MNase versus sonication (B) ChIP-qPCR for single (top) and reChIP (bottom) experiments using IgG (Invitrogen, black), H3K4me3 (CST 9751, green) and H3K27me3 (CST 9733, red) antibodies on sonicated chromatin. An active H3K4me3 region (Gapdh1), inactive H3K27me3 region (Meis2) and 6 bivalent regions are assayed, four of which are novel to this study. (C) Single ChIP-qPCR analysis using IgG (Invitrogen, left), H3K4me3 (Millipore 07-473, middle) and H3K27me3 (Active Motif 91167, right) antibodies with variable number of cells ranging from 1 million (1M), through to 2,000 (2K). Five primer sets were used including three H3K4me3 enriched regions (green) and two H3K27me3 enriched regions (red). Note that below 50K the background increases and specificity of enrichment is no longer detected. For this experiment a different H3K27me3 antibody was used to the sequenced reChIP datasets. (D, E) downsampling analysis for K4-K27 and K27-K4 datasets using the higher coverage replicate 2. Peaks were called separately for each downsampled dataset and classified according to Figure 3A. (D) Total peaks, (E) promoter peaks. (F, G) Downsampling analysis of independent total H3K4me3 and total H3K27me3 from (GSE135841) (7) showing in silico bivalent predictions atall peaks (G) and promoter peaks (H) at different simulated read depths. (H) Pseudocolour density scatterplot showing log2 CPM/bp enrichment at combined H3K4me3 and H3K4me3-IgG peaks for total H3K4me3 single ChIP (x-axis) compared to H3K4me3-IgG reChIP (y-axis). R=0.906. Data reanalysed from Mas et al. 2018 (14). Figure S2. (A) Schematic showing peak calling strategy for in silico bivalent peak prediction (B-F) Genome browser views of (B) H3K4me3-only, (C) high-confidence, (D) K4-biased, (E) K27-biased, and (F) low confidence genes showing H3K4me3 (green), total H3K27me3 (red) and K4-K27 (purple) and K27-K4 (blue) reChIP datasets. Height of peak re [file 13072_2024_527_MOESM2_ESM.zip › Supplementary figures/SupplementalFigure2.pdf]
